# The Impact of Bariatric Surgery on Sleep Disordered Breathing Parameters From Overnight Polysomnography and Home Sleep Apnea Test

**DOI:** 10.7759/cureus.2593

**Published:** 2018-05-08

**Authors:** Saif Mashaqi, Kristine Steffen, Ross Crosby, Luis Garcia

**Affiliations:** 1 Sleep Medicine, Sanford Health; 2 School of Pharmacy/pharmaceutical Sciences, North Dakota State University; 3 University of North Dakota, Neuropsychiatric Research Institute; 4 Bariatric Surgery, University of north dakota

**Keywords:** obstructive sleep apnea, bariatric surgery, roux-en-y gastric bypass, laparoscopic sleeve gastrectomy, apnea-hypopnea index, obesity

## Abstract

Background

Obstructive sleep apnea (OSA) is a common sleep disorder, especially in patients with obesity. Bariatric surgery is an effective tool to reduce weight and treat co-morbid diseases in patients with morbid obesity. One of these disorders is OSA. The most common bariatric procedures currently performed are Roux-en-Y gastric bypass (RYGB) and laparoscopic sleeve gastrectomy (LSG).

Objectives

Our study demonstrates that bariatric surgery is a very effective tool to reduce the severity of OSA, if not resolve it.

Methods

The medical charts of nine patients who had OSA and underwent bariatric surgery (LSG or RYGB) were reviewed and the apnea-hypopnea index (AHI) was compared before and after surgery. The study was conducted at the Sanford sleep center which is affiliated with the University of North Dakota School of Medicine.

Results

One patient was excluded from the statistical analysis since he was the only male patient, the remaining nine female patients had a significant reduction in AHI after surgery. The mean AHI before surgery was 40 events per hour and seven events per hour after surgery (P 0.004). The mean follow-up with sleep study after surgery was 16 months. The mean reduction in AHI was 80%. There was also an improvement in oxygen saturation (SpO2) before and after surgery (90% and 94% respectively, P 0.008).

Conclusion

The study confirms the significant reduction in AHI after bariatric surgery in female patients with OSA especially short term (one to two years postoperatively).

## Introduction

Obesity is a very common disease in the United States (US) and worldwide. It is a challenging epidemic that is faced by the US population. The prevalence of obesity in the US in 2014 was 29% with age-adjusted prevalence being increased progressively from 23% to 35% from 1988 to 2012 [1]. There has been an increase in the prevalence of obesity in females compared to males over the last five to six years [2]. The most common measurement of obesity worldwide is the body mass index (BMI) which is calculated using height in meters and weight in kilograms. Based on this, the National Institutes of Health (NIH) and the World Health Organization (WHO) classified obesity into the following categories: underweight (<18.5 kg/m2), normal weight (between 18.6 and 24.9 kg/m2), overweight (between 25 and 29.9 kg/m2), obesity class I (between 30 and 34.9 kg/m2), obesity class II (between 35 and 39.9 kg/m2), and morbid obesity (=> 40 kg/m2) [3].

Morbid obesity is associated with a higher risk of co-morbid diseases, including metabolic disorders (e.g., hyperlipidemia and diabetes mellitus), cardiovascular disorders (e.g., hypertension, myocardial diseases, cerebrovascular diseases, and venous thrombotic diseases), musculoskeletal disorders (e.g., osteoarthritis and gout), gastrointestinal disorders (e.g., gastroesophageal reflux disease and hepatobiliary diseases), genitourinary diseases, and cancer [4-6]. Sleep-related breathing disorders (e.g., OSA and sleep-related hypoventilation) are very common in patients with morbid obesity and the severity of OSA tends to be correlated with BMI [7]. One of the treatment options for morbid obesity is surgery. Weight loss surgery (i.e., bariatric surgery) is an effective method to treat obesity and decrease the risk of co-morbid conditions related to it. Bariatric surgery is indicated for BMI > 40 Kg/m2 or BMI > 35 kg/m2 with co-morbid illnesses [8]. The most common bariatric procedures performed in the US are the Roux-en-Y gastric bypass (RYGB) and laparoscopic sleeve gastrectomy (LSG) [9]. We conducted this study to examine the impact of weight loss surgery on the apnea-hypopnea index (AHI).

## Materials and methods

We reviewed the medical charts of ten patients (one male and nine females) between the ages of 41 and 66; however, the one male patient was excluded from statistical analysis to avoid skewing of the results. All patients were morbidly obese except one (BMI 39 kg/m2). We conducted overnight polysomnography (PSG) in all patients before and after bariatric surgeries (two patients underwent home sleep apnea test (HSAT) postoperatively). Seven patients underwent RYGB and three underwent LSG. The pre-operative PSGs were conducted at variable time frames from the surgery ranging from three months to six years. The post-operative PSGs and/or HSATs were conducted within 1-2 years after surgery except for one patient who had it four years after surgery. PSG parameters obtained were total sleep time, AHI, rapid eye movement (REM) AHI, periodic limb movement index (PLMI), periodic limb movement arousal index (PLMarI), spontaneous arousal index (SAI), and oxygen saturation (SpO2). The HSAT parameters obtained were (total sleep time, AHI, and SpO2).

PSGs were conducted using Embla Sandman, Version 10.1.1 (Natus Medical Inc., Pleasanton, CA, US). The obstructive AHI was calculated using the total number of obstructive apneas (defined as => 90% reduction of the flow for > 10 seconds) and obstructive hypopneas (using Type 1b rule defined as => 50% reduction in the flow for > 10 seconds with associated drop in SpO2 by => 4%) divided by total sleep time. OSA severity was classified based on the AHI into mild (=> 5 and < 14.9 events per hour), moderate (=> 15 and < 29.9 events per hour) and severe (=> 30 events per hour) [10]. HSATs were conducted using SleepMed Watermark Apnea Risk Evaluation System (ARES), model 610 (Type III) device (ARES operations, SleepMed Inc., West Palm Beach, FL, US) with ten channels. The ARES device does not stage but differentiates wake from sleep and REM from non-rapid eye movement (NREM). Arousals associated with respiratory events are scored based on a change in snoring and head movements. We used the same PSG rules to score apneas and hypopneas. The device applies a patented algorithm to calculate SpO2 and mean SpO2 (m SpO2). All PSGs and HSATs were interpreted by board-certified sleep physicians at the Sanford sleep center.

In addition to the PSG and HSAT measures, we collected the STOP-BANG score which includes loud snoring, tiredness during daytime, observed apnea, history of hypertension, BMI > 35 Kg/m2, age > 50, neck circumference > 16 inches, and male gender. A score of 0-2 is less likely associated with OSA, while a score of => 3 is associated with high risk. The study was approved by the Sanford Health Institutional Review Board (IRB ID STUDY00001036).

## Results

Starting with PSG results, four patients had severe OSA (defined as AHI =>30 events per hour), four patients had moderate OSA (defined as AHI =>15 and < 30 events per hour) and one patient had mild OSA (defined as AHI => 5 and < 15 events per hour) [10]. After surgery, in the severe OSA group, one patient had the AHI normalized, one patient had AHI decreased to the moderate range, and two patients to the mild range. In the moderate OSA group, four patients had AHI normalized and one patient remained in the moderate range but dropped from 23 events per hour to 15 events per hour. In the mild OSA group, one patient had the AHI normalized (Table 1).

Also, we collected REM AHI which is an important PSG parameter that is affected significantly by obesity [11]. Unfortunately, five patients did not have this measure available but in the other four patients, there was a reduction in the REM AHI. Two patients progressed from the mild, moderate, and severe range to the normal range, while the other two patients moved to the mild range. SpO2 improved in all (including the male patient) but one patient (this patient’s SpO2 remained the same). The improvement in SpO2 ranged between 1% and 14% (Table 2). STOP-BANG score was measured in six patients and was => 3 in all of them before surgery and normalized in all of them after surgery (Table 2).

**Table 1:** Mean values of body mass index, polysomnograms, home sleep apnea test measures, and STOP BANG before and after bariatric surgery * P value performed using Wilcoxon signed-rank test. ** STOP-BANG => 3 is associated with high risk of obstructive sleep apnea and < 2 with low risk of obstructive sleep apnea.

Variable	N (cases)	Before surgery (Mean +/- SD)	After surgery (Mean +/- SD)	P value *
BMI (kg/m2)	9	49 +/- 10	30.3 +/- 3.5	0.004
AHI (event/hour)	9	40.6 +/- 36.1	6.9 +/- 7.08	0.004
REM AHI (event/hour)	4	54.5 +/- 12.6	7.9 +/- 4.8	0.13
SPO2 (%)	9	90.4 +/- 4.2	94.2 +/- 1.9	0.008
STOP-BANG **	5	4.8 +/- 1.3	1 +/- 1	0.06

**Table 2:** The apnea-hypopnea index (AHI) before and after bariatric surgery and the percent reduction in AHI in all patients

Case	Age	AHI (Before surgery)	AHI (After surgery)	Follow up (Months)	% reduction in AHI	BMI after bariatric surgery
1	46	48.4	2.6	8	95	29.5
2	59	21	0.0	13	100	30
3	64	10.7	1.2	15	89	33
4	66	17.7	4	18	77	35
5	49	117	21	10	82	33
6	61	23	15.1	52	34	24
7	54	82	8.7	6	89	32
8	45	15.8	2	6	87	31
9	55	30	8	24	73	26
Mean +/SD	55.4 +/- 7.6	40.6 +/- 36.1	6.9 +/- 7.08	16.8 +/- 14.4	80.6 +/- 19.4	30.4 +/- 3.5
Median	55.00	23.00	4.00	13.00	87.00	31.00

## Discussion

Weight loss surgery is a common modality treatment that is used for rapid weight loss and subsequent control of many co-morbidities associated with morbid obesity. One of these co-morbid conditions is OSA. The success of bariatric surgery in treating sleep disordered breathing and specifically obstructive apnea has been defined variably. One definition is a postoperative reduction of AHI by > 50% and AHI < 20 events per hour [12]. Quintas-Neves et al. published a review article summarizing the efficacy of bariatric surgery on OSA. Out of 27 studies, nine studies focused on RYGB and only one study focused on LSG. Eight studies were prospective case series and two studies were retrospective. The sample size was around 50 patients or less in these studies except in one study by Haines et al. where they included 289 patients [13].

The follow-up time after surgery was variable in these studies (ranging between three months and five years) [14]. Some studies suggested that the greatest reduction in AHI after bariatric surgery occurs in the first year, post-op. This is followed in some cases by an increase in the AHI which is independent of BMI, suggesting other factors contributing to OSA relapse in the long-term post-op [15-16]. Other metabolic theories introduced to explain the reduction in AHI, aside from BMI reduction, include decrease in the intra-abdominal pressure with subsequent improvement in the oxygenation to the respiratory centers in the central nervous system [17] and the anti-inflammatory effect of weight loss and the reduction in some inflammatory biomarkers (especially TNF alpha) [18].

It is very hard to determine which bariatric surgery results in the greatest reduction in AHI since these studies had variable preoperative BMI and AHI baselines, different follow-up time, different methodology for the diagnosis of OSA (full overnight PSG vs sleep-related questionnaires), and different inclusion and exclusion criteria "as in our study". This makes it difficult to extrapolate results between these studies.

**Figure 1:**
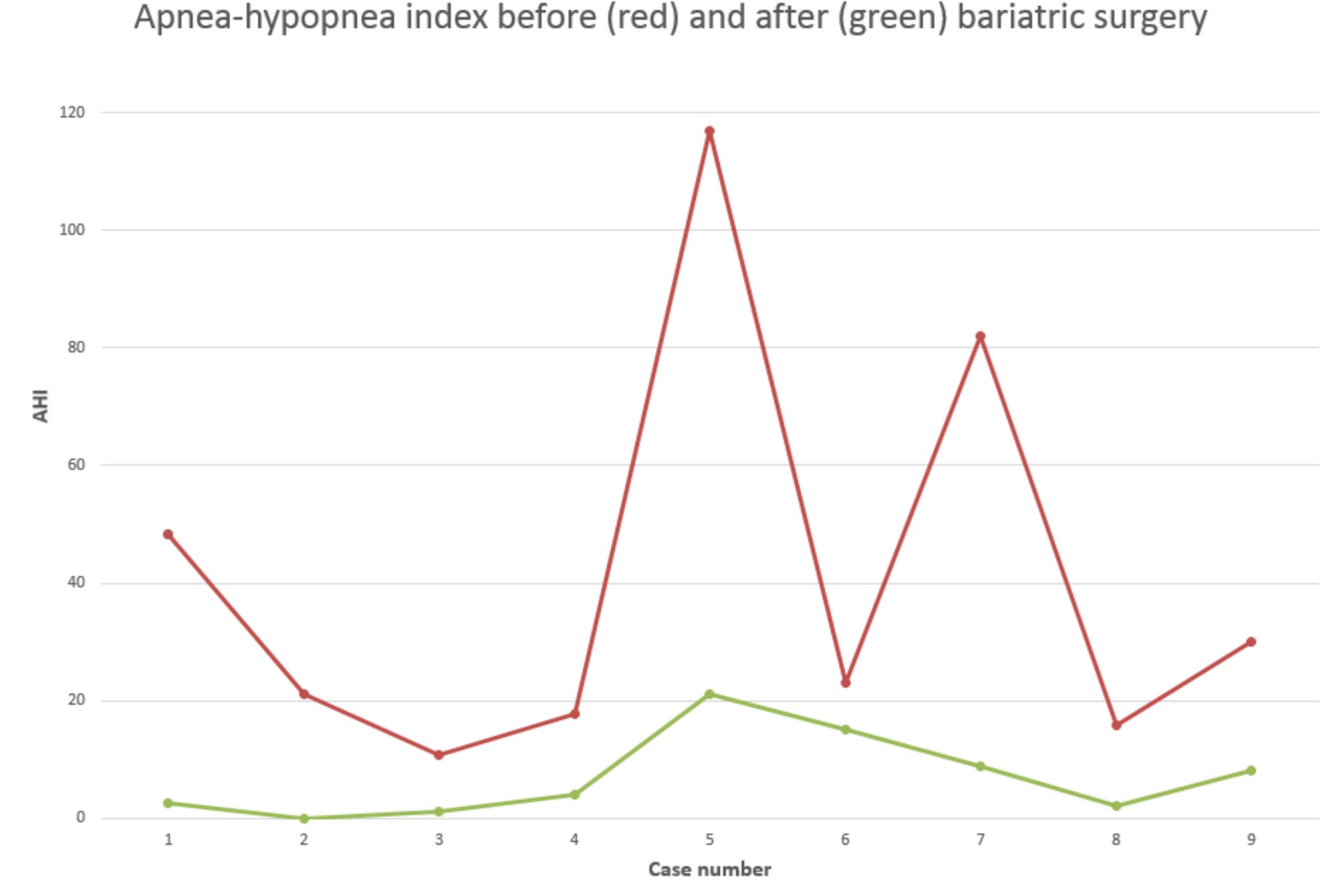
Apnea-hypopnea index before (red) and after (green) bariatric surgery

Our case series results concur with the literature results and the postop “success” definition. There was only one patient who had < 50% reduction in AHI after surgery (Figure 1). This patient had the PSG more than 4 years after surgery which suggests that this patient might have gained weight that resulted in the AHI increase, although, other metabolic factors not related to BMI can be involved. The mean follow-up time in our sample was 16 months which is within one to two years post-op that AHI is expected to drop the maximum. We also showed a reduction in REM AHI (which was not statistically significant but this could be due to a very limited sample size of just five patients) and improvement in SpO2 (Table 1).

Our study had some limitations. First, it is a case series with a small sample size (nine patients). However, as noted earlier, most of the studies in the literature were case series and retrospective studies with few randomized clinical trials [16,19-20]. Second, most of our patients underwent RYGB and only three patients underwent LSG. At our bariatric and metabolic center, RYGB is the most common procedure performed. According to the American Society for Metabolic and Bariatric Surgery (ASMBS), RYGB was the most common procedure in 2012 (37%) compared to LSG (33%) but there has been a switch over in the last few years with LSG being the most common procedure in 2016 (58%) compared to RYGB (19%). Third, unfortunately, we could not obtain the whole PSG parameters pre and post-op (e.g., REM AHI) since two patients had HSAT and three patients had PSG at an outside facility which mandates cautious interpretation of these data. Fourth, all patients are females which can cause a bias in the AHI since OSA tends to be more severe in males. Finally, there were variable times at which the PSGs were obtained pre- and post-bariatric surgery.

## Conclusions

Our study concurs with the body of literature that bariatric surgery (specifically RYGB and LSG) results in a significant reduction (if not normalization) in AHI at least for a short-term post-operation period (the first two years). More randomized clinical trials need to be conducted to confirm these results and explain OSA relapse that is seen in some cases in the long term, especially, if not related to weight re-gain.
